# Horizontal Collectivism Moderates the Relationship Between in-the-Moment Social Connections and Well-Being Among Latino/a College Students

**DOI:** 10.1007/s10903-021-01143-5

**Published:** 2021-01-27

**Authors:** Maryam Hussain, Carmen Kho, Alexandra Main, Matthew J. Zawadzki

**Affiliations:** Department of Psychological Sciences, University of California, Merced, 5200 N. Lake Road, Merced, CA 95343 USA

**Keywords:** Momentary health, Ecological momentary assessment, Hispanic/Latino/as, College students

## Abstract

Sleep problems and poorer well-being may be particularly salient for Latino/a college students as they tend to experience sociocultural adjustments during this transitory time. Social connections, a correlate of health, change moment-to-moment for college students and may be experienced differently for people who more strongly endorse horizontal collectivist cultural values. We used ecological momentary assessment (EMA) to examine how in-the-moment social connections influence in-the-moment health, and how horizontal collectivism moderates the moment-to-moment associations. Self-identified Latino/a college students (*n* = 221) completed a demographic information and cultural values questionnaire and then responded to EMA measures on their social connections, affective and subjective well-being, and sleep for 14 consecutive days. Better in-the-moment social connections associated with better health. Horizontal collectivism moderated some, but not all associations between social connections and health. Social connections are multidimensional and differently predict in-the-moment health among Latino/a college students who more strongly endorse horizontal collectivistic values. We discuss implications for identifying vulnerable well-being moments among this understudied population.

## Background

College students often experience poor sleep [1–3] and mood disorders, such as depression and anxiety [4, 5]. These problems may be especially salient for Latino/a students who often struggle with adjustment in college due to differences between their heritage culture and the dominant culture [6, 7]. Yet, we know little about Latino/a college students’ well-being and sleep as a function of their sociocultural experiences despite this being the fastest growing ethnic group in US colleges [8]. Understanding relationships with well-being and sleep problems is important as they can be detrimental to students’ immediate academic success and cognitive functioning [9, 10] and lifelong morbidity [11]. Thus, the purpose of this paper is to test social and cultural factors that relate to sleep, subjective health, and affective well-being among Latino/a college students.

### Social Connections and Health

We focused on social connections in this study given the importance of social relationships within Latino/a culture [12, 13]. Past studies suggest social connection is multidimensional [14], with each dimension having potential relationships with health-related outcomes. In general, people who report more positive social connections, such as high-quality social interactions, have better health outcomes [15], while those reporting poor social connections, such as loneliness, have worse health [16]. Ecological momentary assessment (EMA) studies indicate that in moments when a person had a social interaction they rated as more pleasant than typical for them [17], or had more social connectedness or less loneliness [18], they had less stress and more positive mood. Yet these social connection dimensions largely have been explored separately, which does not clarify whether simply having more social connections is important or whether particular dimensions uniquely relate to well-being.

Thus, the first goal of this paper is to examine social connections in a multidimensional manner—quality of the interaction, inclusion of other in the self during the interaction, and feeling lonely—and test their relationships with well-being and sleep. Quality of social interaction assesses perceived valence of the interaction with past work indicating this valence component accounting for more variability in predicting positive well-being than simply asking if a person was engaging in a social interaction [17]. Inclusion of other in the self during interaction assesses the perception of being interconnected with an interaction partner [19]. Although we could not identify empirical studies connecting inclusion of other in the self and health, we use evidence from research on social interdependence—a correlate of inclusion of the other in the self [20]. Research on the importance of social interdependence for positive well-being and health suggests that collectivistic cultures, such as Latinos/as, benefit from having social connections that promote interrelational harmony [21]. Finally, loneliness is the perception that one’s social connections are inadequate in meeting one’s social involvement preferences [14]. Feeling lonely has been linked to poorer self-rated health [22], feeling sad [23], and sleep problems [24]. As such, these operationalizations allow a multidimensional approach to testing if one’s momentary social connections relate to health.

### Moderating Effects of Cultural Values

The second goal of this paper is to test whether the cultural value of horizontal collectivism moderates the relationships between momentary social connections and well-being and sleep. Horizontal collectivism is a specific cultural pattern of collectivism focusing on interdependence and social obligations [25, 26], valuing maintaining positive social relationships and time spent with others [27]. Critically, horizontal collectivism is an important value to Latinos/as [28, 29], yet there is variance in endorsement of this cultural value within Latinos/as [26]. In line with the theory of person-environment fit [30], we expect that when a person who endorses horizontal collectivism is not in environments that match this value, such as environments defined by negative or poor social connections then this person will experience worse well-being and sleep outcomes.

Taken together, the present study aims to examine how in-the-moment social connections influence in-the-moment well-being and sleep, and how horizontal collectivism moderates the moment-to-moment associations. We used EMA, a technique that takes repeated brief assessments within and across days as participants go about their daily lives [31], to examine how variation in momentary or daily social connections relates to variation in momentary or daily well-being among Latino/a college students. The benefits of EMA include measurement of psychological processes in real-time in a naturally occurring environment [32], an approach needed with research demonstrating that one’s social environment and well-being can vary from moment to moment [17, 18, 33]. Although EMA is more demanding of participants than global surveys due to the frequency of participation, EMA data provide better and more precise real-time estimates of psychosocial events in daily life. Taking these benefits together, an EMA approach improves the ecological validity of findings [17]. We hypothesized that when Latino/a college students have quality social interactions, feel the other person is included as a part of who they are, and feel less lonely, they will report better well-being and sleep than is typical for them (i.e., poorer subjective and affective well-being, and poorer sleep). We also hypothesized that these associations will be stronger for those who more strongly endorse horizontal collectivism.

## Methods

### Participants

Self-identified Latino/a college students (*n* = 221) participated in the study. The sample was 79.6% female, and aged from 18 to 42 years old (*M* = 19.8, *SD* = 2.51). Most participants indicated they grew up with a primary caretaker that had a high school degree or less (64.3%). See Table 1 for participant information.

**Table 1:** Participant characteristics

Variables	Descriptive information
	*M* (SD) [range]
Age	19.8 (2.51) [18.0–42.0]
Horizontal collectivism	6.65 (1.30) [2.00–9.00]
	*n* (%)
Gender	
Male	45 (20.4%)
Female	176 (79.6%)
Academic classification	
First-year/freshman	85 (38.5%)
Second-year/sophomore	47 (21.3%)
Third-year/junior	34 (15.4%)
Fourth-year/senior	48 (21.7%)
Other	7 (3.2%)
Parental education	
High school or less	97 (43.9%)
High school graduate	45 (20.4%)
Some college	30 (13.6%)
Associate’s/vocational degree	8 (3.6%)
Bachelor’s degree	11 (5.0%)
Other	30 (13.6%)
Total N	221

### Data Collection

Eligible participants were recruited online using a campus-based subject pool system (SONA System) as part of a larger study investigating how individuals experience culture in daily life. The study was conducted in two phases. First, at baseline, participants came to the campus laboratory, provided informed consent, and completed a Qualtrics questionnaire assessing demographic information and cultural values, among other measures not relevant to the present study. Participants were compensated with course credit for the baseline session.

Second, participants were given an option to participate in the EMA portion of the study. From the 221 that participated in the baseline portion, 159 chose to participate in the EMA portion. When prompted via a smartphone app (RealLife Exp, Life Data Corporation, Marion, IN), participants responded to two measures each day for 14 consecutive days that assessed social connections, general well-being, and other items that are not pertinent to this study (duration ~5 min with 31 items). Additionally, participants responded to a morning measure in which sleep duration and quality were assessed, and other items that are not pertinent to this study (duration ~1 min with seven items). Participants did not receive a notification for morning items, rather, they were instructed to respond those items upon waking. Notifications to respond to momentary experiences were semi-randomized, with the first notification occurring randomly 12-4 pm and the second notification between 6 and 10 pm. As missing data are a common problem in EMA research, and the majority of participants will have missing data when repeated assessments are conducted over a period of weeks [32], participants were encouraged to complete at least 80% of the assessments. Indeed, a review of EMA methods in health settings suggests that a 75–80% response rate is common in many studies [31]. For morning data, on average, 94 participants completed at least 90% of the sessions, 141 completed at least 80%, and 157 completed at least 70%. For momentary data, on average, 92 had at least 90% data, 138 had at least 80%, and 155 had at least 70%.

### Measures

#### Horizontal Collectivism

At baseline, participants completed the four-item horizontal collectivism subscale from the Individualism and Collectivism Scale (IACS) [34]. This subscale captures both the cultural orientation of collectivism and the expected affiliated socialization pattern of interdependence (sample item: “I feel good when I cooperate with others”). Participants responded on a 1 (*never/definitely no*) to 9 (*always/definitely yes*) scale. Responses were averaged together such that higher values indicated greater endorsement of horizontal collectivism (α = 0.66).

#### Momentary Social Connections

Participants completed a series of social connection variables via EMA. First, participants were asked “Did you have a social interaction since the last beep?” to assess if a social interaction had occurred since the last prompt. If they indicated ‘no’, then they were not asked more questions about the social interaction (i.e., quality and inclusion of the other in the self). If they indicated ‘yes’, then they were asked to rate from 0 (*very hostile*) to 6 (*very pleasant*) the quality of social interaction with the question “How pleasant was the interaction?” Additionally, if they indicated ‘yes’ to having had a social interaction, participants reported on their perceived closeness of the relationship with the person with whom they had the interaction using the single item Inclusion of Other in the Self Scale [19]. The item was adapted for EMA such that participants viewed an image of two circles with one labeled as ‘self’ and the second circle labeled as ‘other’; participants indicated on a scale of 1 (*circles not touching*) to 7 (*circles almost completely overlapping*) how close they are with that person. Regardless of whether the participant had reported an interaction or not, participants reported on their feelings of loneliness by responding to the following question, “Right now, how lonely do you feel?” on a scale from 0 (*not at all*) to 6 (*extremely*). Almost 84% of the total observations had a social interaction reported, thus we restricted our analyses to moments when a social interaction had occurred. This parameter restriction only affects the loneliness variable, which should be interpreted as how lonely a person felt even during times when a social interaction occurred recently. Suggesting that all three social connection items were capturing different information and should be tested separately, across all observations, correlations ranged from small to small-moderate, *r*s = |0.18| to |0.40|, *p*s < 0.001.

#### Momentary Well-Being

At each observation participants reported their subjective and affective well-being. Subjective well-being was assessed with the following item, “Right now, would you say your health is..?” answered on a 0 (*poor*) to 6 (*excellent*) scale. Affective well-being consisted of two separate items answered on a 0 (*not at all*) to 6 (*extremely*) scale: “Right now, how [sad, anxious] do you feel?”. Correlations across these items suggested small to moderate relationships and thus were analyzed separately, *r*s = |0.36| to |0.54|, *p*s < 0.001.

#### Daily Sleep

Each morning participants reported on their sleep from the night before. To assess sleep duration, participants reported their response in hours and minutes to the following question, “How long did you sleep?” Sleep quality was assessed by the question, “How well did you sleep?” with a response scale of 0 (*not at all*) to 6 (*extremely*). Sleep duration showed a small correlation with sleep quality, (*r* = 0.26, *p* < 0.001), and thus were analyzed separately.

### Analysis Plan

For subjective and affective well-being variables we modeled a two-level structure with momentary observations (Level 1; momentary social connections and well-being) nested within person (Level 2; person-level characteristics); for the sleep variables we modeled days (Level 1; daily social connections and sleep) nested within person (Level 2; person-level characteristics). Multilevel modeling analyses with restricted maximum likelihood were performed using PROC MIXED command in SAS 9.4. The restricted maximum likelihood method does not impute missing data, rather it uses available data to calculate maximum likelihood estimates. This approach is recommended for EMA data as it is robust in addressing missing data, which can often be problematic in repeated measurements [35].

To test goal one of the paper, we conducted separate multilevel models for each outcome where we entered all social connection predictor variables (Level 1) simultaneously into each model. This approach allowed us to explore which social connection variable best predicted the outcome and how its predictability was different from other social connection variables within the model. To test goal two of the paper, we similarly conducted separate multilevel models for each outcome. We entered all social connection variables, horizontal collectivism (Level 2), and interaction terms between social connections and horizontal collectivism into each model. This approach allowed us to test the main focus of goal two: identify which interaction term between horizontal collectivism and each social connection dimension best predicted the outcome and how each interaction term may be different from the other interaction terms within the model.

For subjective and affective well-being as outcomes, social connection dimensions were person-mean centered at the momentary level (Level 1); this approach allowed us to compare a person to their own typical levels of social connections (Level 2). In all interaction models, horizontal collectivism was grand-mean centered (Level 2). Covariates in the models included time (Level 1) and parental education (Level 2). Time comprised study day (ranging from 1 to 14), weekday (0 = Monday to Friday) or weekend (1 = Saturday and Sunday), and time of day (number of minutes elapsed since midnight of each day). Parental education was coded for highest level attained by participant’s primary childhood guardian (0 = high school or less, 1 = more than high school). All predictors were modeled using fixed effects, with a random intercept to account for initial variance between individuals in outcomes. We expected observations closer in time to each other to be more correlated and thus specified an autoregressive covariance structure.

For sleep as an outcome, because it was measured at the day level, social connection dimensions were averaged for each day and then person-mean centered at the day-level. Time was controlled for in terms of study day and weekday/weekend, and parental education was controlled for whether the participant’s parent had at least a high education or not. All predictors were modeled using fixed effects, with a random intercept. An unstructured covariance parameter was indicated for the sleep outcome models as we did not expect constant variability in the observations.

### Ethics

The study was reviewed and approved by the Institutional Review Board at the study site.

## Results

Given the multilevel nature of all models, we calculated a pseudo-*R*^2^ to account for total variance in the outcome (see Tables 2, 3, 4, and 5). This value uses the estimated parameters from the model to create a predicted score of the dependent variable for each moment of data collected, and then correlates the predicted value with the actual value [36]. This statistic should be interpreted as the proportion of the explained variance in the random effect of the conditional model compared to the null model [35, 36].

**Table 2:** Multilevel models of the within-person effects of momentary social connections on subjective and affective well-being

	Subjective well-being	Sadness	Anxiety
*Model statistics*			
Pseudo *R*^*2*^	0.02	0.13	0.06
*Fixed effects*			
Intercept	3.35 (0.16)***	1.25 (0.16)***	1.88 (0.18)***
Study day	0.00 (0.01)	0.00 (0.01)	−0.00 (0.01)
Weekend	0.06 (0.04)	0.03 (0.05)	−0.15 (0.06)**
EMA minutes	0.00 (0.00)	0.00 (0.00)	−0.00 (0.00)
Parent education	0.23 (0.22)	−0.03 (0.18)	0.04 (0.22)
Quality interaction	0.12 (0.02)***	−0.19 (0.03)***	−0.11 (0.03)***
Inclusion of others in self	−0.02 (0.01)	0.05 (0.02)**	0.01 (0.02)
Loneliness	−0.16 (0.02)***	0.48 (0.02)***	0.34 (0.03)***
*Random effects*			
Intercept	1.27 (0.18)***	0.46 (1.73)	1.01 (0.19)***
AR	0.86 (0.05)***	0.98 (0.08)***	0.88 (0.05)***
Residual	0.58 (0.03)***	1.11 (0.04)***	1.23 (0.05)***

*Notes.* All effects are unstandardized beta coefficients with standard errors in parentheses

**p < 0.01, *** *p* < 0.001

**Table 3:** Multilevel models of the within-person effects of daily social connections on sleep

	Sleep duration	Sleep quality
*Model statistics*		
Pseudo *R*^*2*^	0.01	0.01
*Fixed effects*		
Intercept	414.08 (11.14)***	3.65 (0.12)***
Study day	0.33 (0.93)	−0.01 (0.01)
Weekend	15.10 (7.47)*	0.05 (0.07)*
Parent education	6.19 (13.58)	0.07 (0.17)
Quality interaction	−1.59 (3.76)	0.06 (0.04)
Inclusion of others in self	−1.49 (2.58)	−0.01 (0.03)
Loneliness	0.97 (3.49)	−0.07 (0.04)*
*Random effects*		
Intercept	3289.81 (639.51)***	0.69 (0.10)***
Residual	11,568 (538.31)***	1.61 (0.06)***

*Notes.* All effects are unstandardized beta coefficients with standard errors in parentheses

*p < 0.05, *** *p* < 0.001

**Table 4:** Multilevel models of the moderating effect of horizontal collectivism on the within-person effects of momentary social connections on subjective and affective well-being

	Subjective well-being	Sadness	Anxiety
*Model statistics*			
Pseudo *R*^*2*^	0.07	0.15	0.07
*Fixed effects*			
Intercept	3.34 (0.15)***	1.25 (0.16)***	1.89 (0.18)***
Study day	0.00 (0.01)	0.00 (0.01)	−0.00 (0.01)
Weekend	0.06 (0.04)	0.03 (0.05)	−0.14 (0.06)**
EMA minutes	0.00 (0.00)	0.00 (0.00)	−0.00 (0.00)
Parent education	0.28 (0.22)	−0.06 (0.18)	0.03 (0.22)
Horizontal collectivism	0.22 (0.07)**	−0.14 (0.06)*	−0.06 (0.07)
Quality interaction	0.12 (0.02)***	−0.19 (0.03)***	−0.13 (0.03)***
Inclusion of others in self	−0.02 (0.01)	0.06 (0.02)**	0.02 (0.02)
Loneliness	−0.16 (0.02)***	0.48 (0.02)***	0.33 (0.03)***
Horizontal collectivism X Quality interaction	0.00 (0.01)	0.02 (0.02)	−0.01 (0.02)
Horizontal collectivism X Inclusion of others in self	0.00 (0.01)	0.02 (0.01)	0.04 (0.01)**
Horizontal collectivism X loneliness	0.02 (0.01)	−0.01 (0.02)	−0.04 (0.02)*
*Random effects*			
Intercept	1.20 (0.17)***	0.22 (4.21)	1.02 (0.19)***
AR	0.85 (0.05)***	0.99 (0.08)***	0.88 (0.05)***
Residual	0.58 (0.03)***	1.12 (0.04)***	1.23 (0.05)***

*Notes.* All effects are unstandardized beta coefficients with standard errors in parentheses

*p < 0.05, **p < 0.01, *** *p* < 0.001

**Table 5:** Multilevel models of the moderating effect of horizontal collectivism on the within-person effects of daily social connections on sleep

	Sleep duration	Sleep quality
*Model statistics*		
Pseudo *R*^*2*^	0.01	0.01
*Fixed effects*		
Intercept	414.08 (11.16)***	3.65 (0.12)***
Study day	0.38 (0.93)	−0.01 (0.01)
Weekend	915.20 (7.50)*	0.06 (0.07)
Parent education	6.95 (13.63)	0.10 (0.16)
Horizontal collectivism	5.78 (4.99)	0.08 (0.06)*
Quality interaction	−1.23 (3.86)	0.06 (0.04)
Inclusion of others in self	−1.68 (2.67)	−0.01 (0.03)
Loneliness	0.87 (3.51)	−0.07 (0.04)
Horizontal collectivism X Quality interaction	3.92 (2.79)	−0.01 (0.03)
Horizontal collectivism X Inclusion of others in self	1.22 (1.65)	0.01 (0.02)
Horizontal collectivism X loneliness	1.18 (2.69)	0.01 (0.03)
*Random effects*		
Intercept	3307.95 (642.24)***	0.68 (0.10)***
Residual	11,557 (538.41)***	1.61 (0.06)***

*Notes.* All effects are unstandardized beta coefficients with standard errors in parentheses

*p < 0.05, *** *p* < 0.001

### Quality of Social Interactions

As expected, when a person experienced better quality social interactions than typical, they reported better subjective well-being (*b* = 0.12, *SE* = 0.02, *df* = 2636, *p* < 0.001), feeling less sad (*b* = −0.19, *SE* = 0.03, *df* = 2648, *p* < 0.001), and feeling less anxious (*b* = −0.11, *SE* = 0.03, *df* = 2644, *p* < 0.001); see Table 2. Quality of social interactions did not predict change in sleep duration or sleep quality (*p*s > 0.05; see Table 3). Horizontal collectivism did not moderate the relationship between quality of social interaction and any of the outcomes (*p*s > 0.05; see Tables 4 and 5).

### Inclusion of Other in the Self

Unexpectedly, when a person reported more overlap in the inclusion of other in the self than typical, they reported feeling greater sadness (*b* = 0.05, *SE* = 0.02, *df* = 2648, *p* = 0.003). Furthermore, inclusion of other in the self during a social interaction did not predict subjective well-being, anxiety, sleep duration, or sleep quality (*p*s > 0.05; see Tables 2 and 3).

**Fig. 1:**
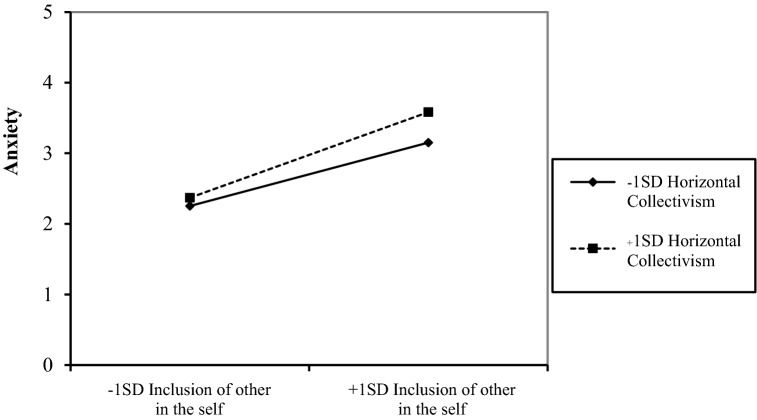
Interaction effect of inclusion of other in the self during an interaction and horizontal collectivism on anxiety

Furthermore, horizontal collectivism moderated the relationship between inclusion of the other in self and anxiety such that when a person who endorsed higher (vs. lower) horizontal collectivism felt more inclusion of other in the self during a social interaction than typical, they reported feeling more anxious (*b* = 0.04, *SE* = 0.01, *df* = 2641, *p* = 0.003; see Fig. 1). Horizontal collectivism did not moderate the relationship between inclusion of other in the self and any of the other outcomes (*p*s > 0.05; see Tables 4 and 5).

### Loneliness

When a person felt lonelier than typical, they reported poorer subjective well-being (*b* = −0.16, *SE* = 0.02, *df* = 2636, *p* < 0.001), more sadness (*b* = 0.48, *SE* = 0.02, *df* = 2648, *p* < 0.001) and more anxiety (*b* = 0.34, *SE* = 0.03, *df* = 2644, *p* < 0.001); see Table 2. On days that a person felt lonelier than typical, they reported poorer sleep quality the next morning (*b* = −0.07, *SE* = 0.04, *df* = 1292, *p* = 0.041). However, feeling lonely did not predict sleep duration (*p* > 0.05; see Table 3).

**Fig. 2:**
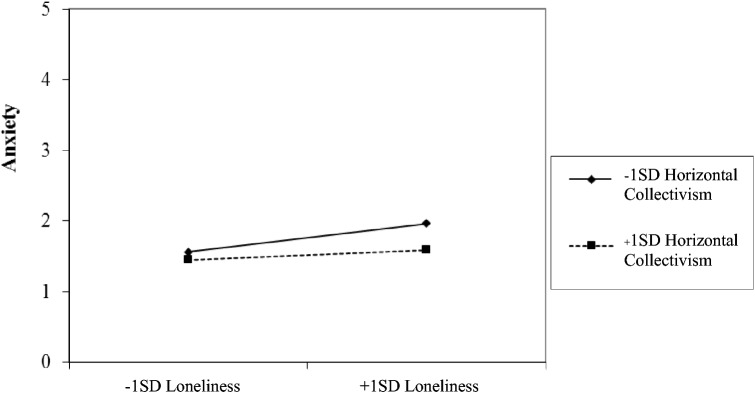
Interaction effect of loneliness and horizontal collectivism on anxiety

Furthermore, when a person who less (vs. more) strongly values horizontal collectivism felt more lonely than typical, they reported feeling more anxious (*b* = −0.04, *SE* = 0.02, *df* = 2641, *p* = 0.026; see Fig. 2). Horizontal collectivism did not moderate the relationship between loneliness and any of the other outcomes (*p*s > 0.05; see Tables 4 and 5).

## Discussion

The first goal of this study was to test our hypothesis that when Latino/a college students have better social connections they will report better well-being and sleep. Our findings indeed provide support for our hypothesis, such that when a person had better quality in-the-moment social interactions they reported better subjective well-being, less sadness, and less anxiety. Conversely, when a person felt more in-the-moment loneliness they reported poorer subjective well-being, more sadness, and feeling more anxious. These findings support within-person EMA research indicating that positive social connections are associated with better well-being outcomes [17, 18]. Furthermore, these findings contribute to the cognitive and stress-buffering model of understanding the role of social relationships in health and wellness during life transitions [37], such as college. That is, when a person interprets social connections with others as meaningful, valuable, and befitting of their social relationship needs, it can be protective against health risks that might otherwise present themselves during moments of transition (e.g., college).

Only one of the social connection dimensions predicted sleep quality. On days when a person reported feeling lonelier than was typical for them, they indicated having poorer sleep quality. This finding supports the idea that when social connections are inadequate in aligning with social connection preferences, it can be detrimental to health and well-being [22–24]. However, we must acknowledge that none of the other social connection dimensions predicted either of the sleep outcomes. Although we predicted aggregated daily social connections would predict sleep duration and quality, we speculate that the immediate effects of daytime social connections may have dissipated by the time the participant went to sleep. Whereas, participants responded to subjective and affective well-being in the same moments they reported on their social connections, they did not report on sleep until the next morning. As such, there was a greater lag in time between reporting social connections and sleep. Furthermore, participants may have experienced other social connections between the last EMA and the time they went to bed that we did not capture. These ‘missed measures’ of social connections may have more of an immediate impact on the duration and quality of sleep. Indeed, one study suggests that social rejection before bedtime, compared to a neutral task, predicted delay in sleep time and fewer hours of sleep [37]. Thus, we speculate that it may be especially important to examine how social connections right before bedtime, rather than an aggregate of all day-time social connections, may influence to sleep duration and quality.

Interestingly, one of our findings suggests that when a person had an interaction with someone with whom they had more inclusion of other in the self, they reported feeling sadder. We draw upon the bidirectional nature of the data to speculate upon this association. Because participants responded to social connection and health items simultaneously, it is possible that in moments that a person felt sad, they purposely engaged in an interaction with someone with whom they felt enmeshed. Indeed, research suggests that people seek social connections when feeling vulnerable (e.g., [37, 38]). We expand on this more in the limitations section. Social interdependence theory supports the idea that Latinos/as in particular may seek social connections that highlight interconnectedness [21], such as inclusion of the other in the self. Future research should examine vulnerability in affective well-being within the context of interdependent social connections. More generally, these findings suggest that there are many ways to measure the social connections or interactions, and they should not be conflated or treated as interchangeable. That is, the quality of a social interaction is not equivalent to how enmeshed with self the person sees the other. Furthermore, these findings provide empirical support for how moment-to-moment variations in social connections relate to changes in health within the same person [17].

The second goal of this paper was to test whether horizontal collectivism moderated the relationships between momentary social connections and well-being and sleep. We found that for the most part, horizontal collectivism did not moderate the effect of social connections on subjective or affective well-being or sleep. Indeed, controlling for the strength of endorsement of horizontal collectivism, having good social connections was important for most people. However, results showed a couple exceptions. Data indicated that when Latino/a college students who more strongly endorse horizontal collectivism feel lonelier than typical they report lower levels of anxiety than those who less strongly endorse horizontal collectivism. However, when they feel more of an inclusion of the other in the self, they report higher levels of anxiety than those who endorse collectivism less. These findings support our hypothesis and the subjective person-environment fit theory [30, 39]. We speculate that because horizontal collectivism focuses on valuing others and, more specifically, maintaining pleasant and meaningful social relationships [25–27], a person who has a deeper connection to that ideology may be less impacted by momentary changes in poorer social connections (i.e., loneliness), but more impacted by momentary changes in social connections that underscore concepts of interdependence and cohesion.

As mentioned earlier, most interactions were non-significant. We speculate that theoretically horizontal collectivism captures aspects of social connections; however, it may exclude other pertinent social features of collectivist cultural values, such as *simpatia* that may be relevant to understanding social relationships among US Latino/as. *Simpatia* emphasizes politeness and having positive interpersonal interactions, especially with friends and family members [40]. Future research examining the link between social interactions and health as a function of pertinent cultural values should incorporate with whom the interaction is occurring (e.g., close friend or family member) to better identify both *who* is most vulnerable to the effects of poor-quality social interactions and *when* those vulnerable moments occur.

### Limitations

We elaborate upon three major limitations to this study. First, we were not able to establish directionality or causality, as the associations between the repeated measures were correlational. Although we interpret our findings framed in the context of past literature that underscore social connections influencing health outcomes, we would be amiss to not speculate that health influences social connections. For example, when a person feels healthier, they may be more likely to seek quality social interactions. Thus, we urge future research to assess more frequent measurements throughout the day so that lagged models can determine temporal directionality between social connections and health. Second, although the purpose of our study was to focus solely on Latino/a college students, inclusion of other ethnic groups who value horizontal collectivism, such as Asian Americans [29] could allow for a comparison between-groups to examine how cultural values link to health in another ethnic group that experiences culture similarly [41]. Past research has shown that Latino/a Americans and Asian Americans often align in overarching collectivistic values when compared to other ethnic groups [42]; however, interdependence may be more differentiated for Asian Americans than Latino/a Americans [29]. Finally, a majority of the respondents identified as female. Some research has suggested that Latinas more strongly endorse the social aspects of collectivistic cultural values than their male counterparts [43–46]. Exploring the potential moderating effect of gender when considering the link between social interactions and health as a function of cultural orientation would be possible by having a more balanced gender ratio.

### New Contribution to the Literature

The major contribution of the present study is our in-the-moment measurement of social connection experiences and health, an extension of work that has just started to emerge on identifying when people are vulnerable to health problems [17]. EMA provided a more detailed assessment of Latino/a college students everyday lives over time, allowing us to identify *when* Latino/a college students feel most vulnerable to adverse health outcomes. Due to the intensive longitudinal data, the seemingly small effects represent consistent findings across *all* measured occasions. Thus, these results could be interpreted as effects that likely accumulate over time, a concept that is explored within the everyday stress pileup model [47]. As such, these findings are a necessary step to future development of just-in-time adaptive interventions [48] aimed at providing the appropriate type and amount of social interaction at vulnerable moments, based on the person’s environment or internal conditions, within the natural environment.

This study was conducted at a Hispanic serving institution, a designation that is given to higher education institutions that enroll at least 25% Hispanic/Latino/a full-time undergraduate students. Past research on health in college students have chiefly occurred at predominantly non-Hispanic White institutions. This overarching environment allowed us to explore at how inherent cultural values interact with moment-to-moment social connections to predict changes in health. As Hispanic/Latino(a) students increasingly represent the ethnic demographics of U.S. universities, it becomes incumbent to understand how college students’ health fluctuates as a function of sociocultural influences.
